# Downregulation of GNA15 Inhibits Cell Proliferation *via* P38 MAPK Pathway and Correlates with Prognosis of Adult Acute Myeloid Leukemia With Normal Karyotype

**DOI:** 10.3389/fonc.2021.724435

**Published:** 2021-09-06

**Authors:** Mengya Li, Yu Liu, Yajun Liu, Lu Yang, Yan Xu, Weiqiong Wang, Zhongxing Jiang, Yanfang Liu, Shujuan Wang, Chong Wang

**Affiliations:** ^1^Department of Hematology, The First Affiliated Hospital of Zhengzhou University, Zhengzhou, China; ^2^Department of Orthopaedics, Rhode Island Hospital, Warren Alpert Medical School, Brown University, Providence, RI, United States

**Keywords:** acute myeloid leukemia, GNA15, cell proliferation, P38 MAPK, prognosis

## Abstract

**Background:**

The prognosis of acute myeloid leukemia (AML) with a normal karyotype is highly heterogonous, and the current risk stratification is still insufficient to differentiate patients from high-risk to standard-risk. Changes in some genetic profiles may contribute to the poor prognosis of AML. Although the prognostic value of G protein subunit alpha 15 (*GNA15*) in AML has been reported based on the GEO (Gene Expression Omnibus) database, the prognostic significance of *GNA15* has not been verified in clinical samples. The biological functions of *GNA15* in AML development remain open to investigation. This study explored the clinical significance, biological effects and molecular mechanism of *GNA15* in AML.

**Methods:**

Reverse transcription-quantitative polymerase chain reaction (RT-qPCR) was used to detect the mRNA expression level of *GNA15* in blasts of bone marrow specimens from 154 newly diagnosed adult AML patients and 26 healthy volunteers. AML cell lines, Kasumi-1 and SKNO-1, were used for lentiviral transfection. Cell Counting Kit-8 (CCK8) and colony formation assays were used to determine cell proliferation. Cell cycle and apoptosis were analyzed by flow cytometry. The relevant signaling pathways were evaluated by Western blot. The Log-Rank test and Kaplan-Meier were used to evaluate survival rate, and the Cox regression model was used to analyze multivariate analysis. Xenograft tumor mouse model was used for *in vivo* experiments.

**Results:**

The expression of *GNA15* in adult AML was significantly higher than that in healthy individuals. Subjects with high *GNA15* expression showed lower overall survival and relapse-free survival in adult AML with normal karyotype. High *GNA15* expression was independently correlated with a worse prognosis in multivariate analysis. Knockdown of *GNA15* inhibited cell proliferation and cell cycle progression, and induced cell apoptosis in AML cells. *GNA15*-knockdown induced down-regulation of p-P38 MAPK and its downstream p-MAPKAPK2 and p-CREB. Rescue assays confirmed that P38 MAPK signaling pathway was involved in the inhibition of proliferation mediated by GNA15 knockdown.

**Conclusions:**

In summary, *GNA15* was highly expressed in adult AML, and high *GNA15* expression was independently correlated with a worse prognosis in adult AML with normal karyotype. Knockdown of *GNA15* inhibited the proliferation of AML regulated by the P38 MAPK signaling pathway. Therefore, *GNA15* may serve as a potential prognostic marker and a therapeutic target for AML in the future.

## Introduction

Acute myeloid leukemia (AML) is a group of heterogeneous clonal hematopoietic progenitor cell neoplasm and the adverse-risk subjects experience poor prognosis (1, 2). Advances in the therapy of AML, such as risk stratification, concurrent chemotherapy, novel drugs and hematopoietic stem cell transplantation, have improved the treatment outcomes of patients (3). Cytogenetic analysis has proved to be crucial for the risk stratification of AML patients (4). However, nearly half of AML patients have a normal karyotype (NK) (4). Identification of the changes in genetic profiles such as mutations of *NPM1*, *FLT3-ITD* and *CEBPA* has further improved the risk stratification of NK-AML. However, most NK-AML still belongs to the intermediate prognostic subgroup in which the most appropriate treatment remains to be defined (4). It is urgent to identify novel prognostic biomarkers and update the risk stratification for NK-AML.

Our group screened and verified 7 new biomarkers of B cell acute lymphoblastic leukemia (B-ALL) based on the ImmuSort database and clinical specimens. G protein subunit alpha 15 (*GNA15*) is one of the prominent candidates (5). In the process of verification, we found that the expression level of *GNA15* in AML was significantly higher than that in B-ALL and normal controls, suggesting that *GNA15* may play an important biological role in AML.

The *GNA15* gene is located on chromosome 19p13.3, spans approximately 27.7 kb, and encodes a 44 KDa GNA15 protein (6). *GNA15* is a member of the *GNA* gene family (including *GNAq*, *GNA11* and *GNA14*) (7). The protein encoded by the *GNA15* gene is the alpha subunit of the G protein, which participates in the regulation of cell proliferation and apoptosis (8). The GNA15 protein, or Gα15, belongs to the Gαq subfamily and is highly expressed in some cell types, such as hematopoietic stem cells and epithelial cells during specific stages of differentiation. It is up-regulated in CD34^+^ hematopoietic stem cells and decreases with cell maturation (9, 10). G protein-coupled receptors (GPCRs) are the largest cell surface molecule family involved in signal transmission. It accounts for more than 2% of genes encoded in the human genome (11). Abnormal activation of GPCRs is related to tumor occurrence and metastasis (12). By coupling to GPCRs, *GNA15* promotes cell proliferation and inhibits cell apoptosis by regulating downstream signaling pathways (10). *GNA15* is known to play as an oncogene in several tumors, such as gastroenteropancreatic neuroendocrine neoplasia (GEP-NEN) (8), liver cancer (13, 14), pancreatic ductal adenocarcinoma (15) and ovarian cancer (16). These biological functions are mediated by ERK, NFκB and Akt signaling pathways in GEP-NEN (8, 13–16). Nevertheless, the clinical value and biological function of *GNA15* in AML remain unknown.

Therefore, in this study, we examined the expression and prognostic value of *GNA15* in AML and its effects on cellular proliferation, cell cycle and apoptosis. The underlying mechanism of *GNA15* regulation was also explored.

## Materials and Methods

### Database Analyses

We collected the expression data of 263 hematopoietic stem cell (HSC) samples and 113 leukemia stem cell (LSC) in the ImmuCo database (http://immuco.bjmu.edu.cn), 74 peripheral blood mononuclear cell (PBMC) samples, 542 AML samples in the ONCOMINE database (https://www.oncomine.org) and 173 AML samples in the Gene Expression Profiling Interactive Analysis (GEPIA2) database (http://gepia2.cancer-pku.cn), and compared the expression level of *GNA15*. One publicly available cytogenetically normal AML data sets (GSE12417 of GEO database) were used to perform survival analysis.

### Subjects

Bone marrow samples from 154 newly diagnosed AML patients (patients with acute promyelocytic leukemia were excluded) and 26 healthy controls were obtained following informed consent at the First Affiliated Hospital of Zhengzhou University between February 2017 and April 2019. Details of treatment regimens were reported (17). Subjects were followed up until death, loss to follow-up or April 2021. Complete remission, relapse, risk stratification, overall survival (OS) and relapse-free survival (RFS) were defined as described (17). The study was approved by the Ethics Committee of the First Affiliated Hospital of Zhengzhou University and informed consent was obtained according to the Declaration of Helsinki.

### Next-Generation Sequencing

The mutational hotspots of genes were assessed by next-generation sequencing. The detection was performed utilizing a Rightongene AML/MDS/MPN Sequencing Panel (Rightongene, Shanghai, China) on an Illumina MiSeq System (Illumina, San Diego, CA) high-throughput sequencing platform. The original data after sequencing was analyzed by bioinformatics using NCBI, CCDS, dbSNP (v138), COSMIC, human genome database (HG19) and other databases to determine the pathogenic mutation site. The average depth of the sequencing was 4837.978Kb, detection sensitivity was ~ 5%.

### Cell Lines and Reagents

The human AML cell lines (Kasumi-1, SKNO-1, and HL-60), were purchased from American Type Culture Collection (Manassas, VA, USA). The human ALL cell lines (Sup-B15, BV173, NALM-6, and BALL-1) were obtained from Guangzhou Jennio Biotech Co. Ltd (Guangzhou, China). The human AML cell line HEL, chronic myeloid leukemia cell line K562, the human lymphoma cell line Ramos and the human myeloma cell line KM-3 were kind gifts from Professor K. Y. Liu of Peking University People’s Hospital. These cell lines were maintained in RPMI 1640 medium containing 10% fetal bovine serum (FBS), 1% streptomycin and penicillin (all from Gibco, Billings, MT, USA) at 37 °C with 5% CO_2_. Cell viability was observed daily with a microscope (Olympus, Ckx53sf, Tokyo, Japan), cell lines (except SKNO-1 cell line) were passaged every 2–3 days and SKNO-1 cells were passaged every 3-4 days. Asiatic Acid (Selleck Chemicals, Houston, TX, USA), was used as a P38 MAPK activator.

### Lentiviral Transduction

Kasumi-1 and SKNO-1 (*FLT3*-wildtype) cells were infected with human GNA15 shRNA lentiviral particles (Genechem, Shanghai, China) or empty control lentiviral particles (Genechem) at a 100 multiplicity of infection (MOI). Media containing lentiviral particles were replaced with a complete medium 12 hours post-infection. Stably transfected Kasumi-1 and SKNO-1 cells were selected with 2 μg/ml and 5 μg/ml puromycin dihydrochloride (Genechem, Shanghai, China) respectively at 72 h post-infection. After 3 weeks of antibiotic selection, stable GNA15-knockdown cells and control cells were obtained. GNA15 expression levels were confirmed by real-time quantitative PCR (RT-qPCR) and Western blot analyses.

### RNA Extraction and RT-qPCR

Bone marrow was collected by Ethylene Diamine Tetraacetic Acid (EDTA) anticoagulant tube and mononuclear cells were isolated from bone marrow by density gradient centrifugation. TRIzol^®^ (Invitrogen, Carlsbad, CA, USA) was used to extract total RNA following the manufacturer’s instructions. The cDNA templates were synthesized with a High Capacity cDNA Reverse Transcription Kit (Applied Biosystems, Foster City, CA, USA) (18). Gene transcript levels were determined by the Taqman method as previously reported (19). Serial dilutions of plasmids expressing *GNA15* and *ABL1* (Genechem) were amplified to construct standard quantification curves. Copy numbers of *GNA15* and *ABL1* were calculated from standard curves using Ct values. Samples were assayed in duplicates to evaluate data reproducibility. The primers and probe sequences of *GNA15* and *ABL1* are shown in **Supplementary Table 1**.

### Western Blot Analyses

RIPA lysis buffer (Solarbio, Beijing, China) with Protein phosphatase inhibitor (Biomed, Beijing, China) and phenylmethylsulfonyl fluoride (PMSF, Biomed) was used to extract total protein lysate. Lysates were run on 10% SDS-PAGE gels, and protein bands were transferred to 0.45μm polyvinylidene difluoride (PVDF) membrane (Millipore, Billerica, MA, USA), then blocked with 5% skim milk at room temperature for 1 hour. The membrane was incubated with primary antibodies (GNA15, Novus Biologicals, Centennial, USA, 1:1000; GAPDH, Solarbio, 1:1000; p-P38 MAPK, P38 MAPK, p-CREB, CREB, p-MAPKAPK2^Thr222^, MAPKAPK2^Thr222^, p53, cleaved-PARP, cleaved-Caspase3, p27 Kip1, Cyclin D1, CDK4, p-p44/42 MAPK, p-AMPKα, p-Akt, p-Smad3, LC3A/B, Cell Signaling Technology [CST], MA, USA, 1:1000) overnight at 4°C and probed with secondary antibodies (goat anti-rabbit IgG, goat anti-mouse IgG, Solarbio, 1:1000) at room temperature for 1 hour. The immunoreactive bands were defined using Super ECL Prime (US EVERBRIGHT, Suzhou, China).

### Cell Proliferation and Colony-Forming Assay

The stably transfected cells were seeded in 96-well plates at a density of 1×10^5^ cells/ml. Then 10 μl of Cell Counting Kit-8 (CCK8, Dojin Laboratories, Kumamoto, Japan) was added into each well after 0, 24, 48, 72 hours. Cell proliferation was detected by a full-wavelength microplate reader at 450 nm 3 hours later. To analyze the colony formation, cells were seeded into 35 mm dishes (6×10^3^ cells/well) in methylcellulose-based MethoCult medium (STEMCELLTM TECHNOLOGIES, Vancouver, British Columbia, Canada). The colonies with ≥50 cells were counted with an inverted microscope after 10 days of growing in a humid incubator. The experiments were performed three times to show the repeatability of the data.

### Cell cycle and Apoptosis Analyses

Cells were seeded into 6-well plates (1×10^5^ cells/ml) and starved by adding serum-free medium for 24 hours to research synchronization, and a complete medium was then added for an additional 48 hours. The Cell Cycle and Apoptosis Kit (US EVERBRIGHT, Suzhou, China) was used for cell cycle analyses and the Annexin V-APC/PI Apoptosis Kit (US EVERBRIGHT) was used as the apoptosis assay. The cell cycle and apoptosis were determined by flow cytometry according to the manufacturer’s instructions.

### Xenograft Tumor Mouse Model

Male 6-week-old BALB/c nude mice (Beijing HFK Bioscience Co., Ltd.; Beijing, China) were prepared for generating xenograft models. All mice were divided into two groups (CTRL and KD, 5 mice/group) with intraperitoneal injections of cyclophosphamide (100mg/kg/d × 2d). After two days, the cells (1.5 × 10^7^ cells suspended in 100 μl PBS) with lentiviral particles were syringed in the right flank. We measured the diameters (the longest and shortest diameters) and calculated the volume (L×I^2^×0.5; L: the longest diameter, I: the shortest diameter) of tumor every other day for 12 days, after that all mice were euthanized and tumor tissues obtained for the following assay. All studies were approved by the Ethics Committee of the First Affiliated Hospital of Zhengzhou University.

### Statistical Analyses

Differences across groups were compared using the Pearson Chi-square test or Fisher exact analysis for categorical data and Mann-Whitney *U*-test or Student’s *t*-test for continuous variables. The Kaplan-Meier method and Log-Rank test were used for survival analysis. A Cox proportional hazard regression model was used to determine associations between *GNA15* transcript levels and OS and RFS. Variables with *P* < 0.1 in the single variable analysis were included in the model. A two-sided *P* < 0.05 was considered significant. Analyses were performed by SPSS software version 26.0 (Chicago, IL, USA) and Graphpad Prism™ 8.01 (San Diego, California, USA).

## Results

### The *GNA15* Gene Is Highly Expressed in Subjects With AML

**Figure 1:**
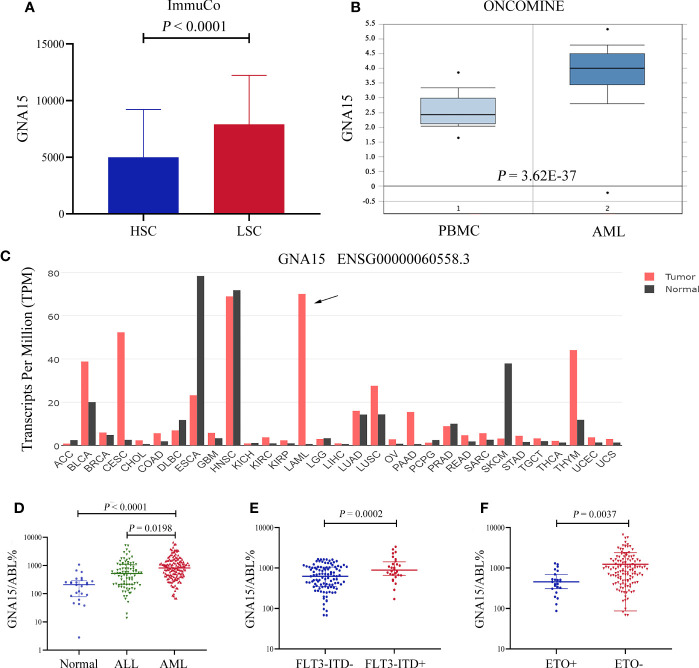
The *GNA15* gene was highly expressed in subjects with acute myeloid leukemia (AML). **(A)** In the ImmuCo database, the gene expression level of *GNA15* was higher in leukemic stem cells (LSC, n=113) than in hematopoietic stem cells (HSC, n=263). **(B)** In the ONCOMINE database, *GNA15* showed significantly higher expression in AML patients (n=542) than in normal peripheral blood mononuclear samples (PBMC, n=74). **(C)** The expression of *GNA15* in various cancers and normal tissues was significantly different and *GNA15* was overexpressed in AML in the GEPIA2 database. The height of the bar represents the median expression of *GNA15* of a certain tumor or normal tissue. **(D)** *GNA15* transcript levels in normal volunteers, acute lymphoblastic leukemia (ALL) and AML. **(E)** *GNA15* transcript levels in AML with or without *FLT3-ITD* mutation. **(F)** *GNA15* transcript levels in AML with or without *AML1-ETO* fusion gene. Error bars indicate median with interquartile range **(D–F)**.

Firstly, we surveyed the expression level of *GNA15* in AML based on the database. As shown in **Figure 1**, the gene expression level of *GNA15* was higher in leukemic stem cells than in hematopoietic stem cells based on the ImmuCo database (*P*<0.0001, **Figure 1A**). *GNA15* expression in AML patients from the Oncomine database was also compared, and similarly, *GNA15* showed significantly higher expression in AML patients than normal peripheral blood mononuclear samples (PBMC, *P*=3.62×10^-37^, **Figure 1B**). Furthermore, by using the public GEPIA2 database, we also observed elevated *GNA15* expression levels in AML (**Figure 1C**). Thus, we surveyed the transcript levels of *GNA15* in the bone marrow of newly diagnosed subjects with AML and normal healthy volunteers. The expression of *GNA15* in AML was significantly higher than healthy volunteers and ALL in our cohort (median 810.00%, range [67.87%-6630.48%] *vs* 209.15% range [0-1073.74%], *P*<0.0001; 810.00%, range [67.87%-6630.48%] *vs* 520.55% range [13.64%-5205.27%], *P*=0.0003; **Figure 1D**). AML with *FLT3-ITD* mutation had higher *GNA15* transcript levels than those without *FLT3-ITD* mutation (889.85% [171.59%-3351.42%] *vs* 625.16% [67.87%-1656.21%], *P*=0.0002, **Figure 1E**). AML with *AML1-ETO* fusion gene had lower transcript levels than those without *AML1-ETO* fusion gene (456.58% [86.78%-1306.69%] *vs* 941.28% [67.87%-6630.48%], *P*=0.0037; **Figure 1F**). The transcript levels of *GNA15* showed no difference in AML with different French-America-British (FAB) classification or cytogenetic risk stratifications (**Supplementary Figure 1**).

### *GNA15* Transcript Levels Are Independently Associated With OS and RFS in AML With Normal Karyotype

**Figure 2:**
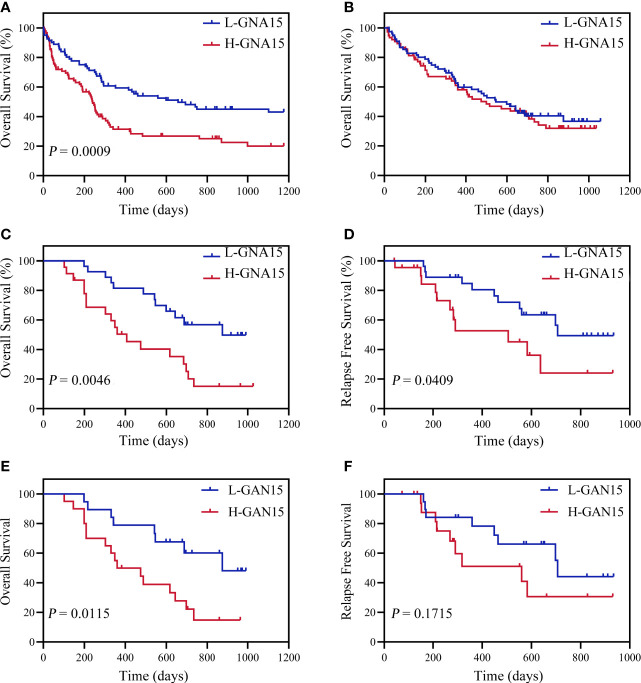
Overall survival (OS) and relapse free survival (RFS) of adult subjects with AML according to *GNA15*. **(A)** OS of 163 AML subjects with normal karyotype in GSE12417. **(B)** OS of 154 AML subjects in our cohort. **(C, D)** OS **(C)** and RFS **(D)** of 50 AML subjects with normal karyotype in our cohort. **(E, F)** OS **(E)** and RFS **(F)** of 39 *FLT3-ITD*-negative subjects in NK-AML cohort according to *GNA15*. H-*GNA15*, high *GNA15* expression; L-*GNA15*, low *GNA15* expression.

Subjects in the GSE12417 database (AML with normal karyotype) were divided into the high expression group and the low expression group according to the median expression value of *GNA15*. Subjects with high expression of *GNA15* showed a relatively worse 3-year OS than the subjects with low expression of *GNA15* (20.3% [10.1%-30.5%] *vs* 43.1% [31.5%-54.7%], *P*=0.0009, **Figure 2A**) in the GSE12417 database. In our AML cohort, AML patients were classified into the high expression group and the low expression group according to the median value of *GNA15* (810.00%). The median follow-up of these 154 patients was 410 days (range 14–1057 days). In the whole cohort, the 2-year OS between low expression group and high expression group showed no significant difference (40.4%, 95% confidence interval (CI) [28.8%-52.0%] *vs* 38.2% [26.2%-50.2%], *P*=0.564, **Figure 2B**). Then we analyzed the prognostic value of *GNA15* in the subgroup with normal karyotype (NK, n=50). In the NK-AML subgroup, subjects in the high expression group were older,had higher expression of Wilms tumor gene 1 (*WT1*) at diagnosis and higher minimal residual disease (MRD) levels after a course of induction chemotherapy (**Table 1**). However, there was no significant correlation between the level of *GNA15* expression and other clinical characteristics, including risk groups, gender, platelets, hemoglobin level, gene mutations and transplant (**Table 1**). In the NK-AML subgroup, subjects with high *GNA15* expression showed a worse 2-year OS and RFS than subjects with low *GNA15* expression (OS: 15.1% [0%-30.8%] *vs* 49.7% [28.1%-71.3%], *P*=0.0046; RFS: 24.1% [0%-49.8%] *vs* 49.4% [26.7%-72.1%], *P*=0.0409; **Figures 2C, D**). Moreover, in the NK-AML without *FLT3-ITD* mutations, subjects with high *GNA15* expression showed a worse 2-year OS (13.7% [0%-50.7%] vs 44.5% [18.2%-70.8%], *P*=0.0115; **Figure 2E**) and a similar 2-year RFS (32.3% [0%-65.0%] vs 40.6% [13.6%-67.6%], *P*=0.1715; **Figure 2F**). In multivariate analysis, high *GNA15* expression was independently associated with a worse OS and RFS (**Table 2**). Female was independently associated with a better OS and RFS (**Table 2**). Other factors, such as age, risk group, *WT1* expression, *FLT3-ITD* mutation and other gene mutations did not show any significant correlation with OS or RFS.

**Table 1:** Relationship between Transcription Level of GNA15 and Clinical Characteristics in Normal Karyotype AML.

Variables	Total	L-*GNA15*	H-*GNA15*	*P*-value
	N = 50	N = 27	N = 23	
Age,years				
Mean ± SD	43.9 ± 15.8	39.4 ± 15.5	49.3 ± 14.7	0.024
≥60, n (%)	7 (14)	2 (7.4)	5 (21.7)	0.295
Female, n (%)	28 (56.0)	16 (59.3)	12 (52.2)	0.615
Risk group, n (%)				0.868
Low	10 (20)	6 (22.2)	4 (17.4)	
Medium	20 (40)	10 (37.0)	10 (43.5)	
High	20 (40)	11 (40.7)	9 (39.1)	
PLT, ×10^9^/l	38 (4-260)	29 (6-260)	45 (4-218)	0.453
Hb, g/l	85.3 ± 21.7	82.5 ± 19.4	88.5 ± 24.2	0.339
Blast of BM (%)	75.0 (0-99)	75.0 (0-99)	75.5 (0-94)	0.016
MRD of flow cytometry (%)	0.7 (0-75.5)	0.9 (0-26.9)	0.9 (0-75.5)	0.003
Mutations, n (%)				
FLT3-ITD	11 (22)	5 (18.5)	6 (26.1)	0.520
NPM1	6 (12)	3 (11.1)	3 (13.0)	1.000
CEBPA	5 (10)	3 (11.1)	2 (8.7)	1.000
DNMT3A	5 (10)	4 (80)	1 (20)	0.449
WT1	6.2 (0-86.9)	0.5 (0-12.9)	6.2 (5-86.9)	0.000
TET2	19 (38)	12 (63.2)	7 (36.8)	0.309
IDH1/2	8 (16)	7 (87.5)	1 (12.5)	0.092
RUNX1	2 (4)	1 (50)	1 (50)	1.000
Transplant, n (%)	11 (22)	8 (29.6)	3 (13.0)	0.285

H-GNA15, high GNA15 expression; L-GNA15, low GNA15 expression; SD, standard deviation; PLT, platelet; Hb, hemoglobin; BM, bone marrow; MRD, minimal residual disease.

**Table 2:** Univariate and Multivariate Analysis of OS and RFS in AML with Normal Karyotype.

End point	Variables	Univariate		Multivariate	
		HR (95% *CI*)	*P*-value	HR (95% *CI*)	*P*-value
**OS**					
	H-*GNA15*	2.77 (1.33-5.79)	0.007	2.62 (1.25-5.51)	0.011
	Age≥60 years	1.74 (0.74-4.07)	0.205		
	Female	0.43 (0.21-0.89)	0.024	0.46 (0.22-0.96)	0.039
	Risk group				
	Low *vs* High	0.62 (0.20-1.90)	0.400		
	Medium *vs* High	0.86 (0.40-1.85)	0.694		
	MRD	1.00 (0.99-1.03)	0.676		
	FLT3-ITD+	0.76 (0.31-1.87)	0.553		
	WT1	1.00 (0.99-1.02)	0.660		
	NPM1	0.96 (0.33-2.74)	0.933		
	DNMT3A	0.86 (0.26-2.86)	0.810		
	IDH1/2	0.82 (0.32-2.16)	0.693		
	TET2	1.36 (0.66-2.81)	0.399		
	RUNX1	3.71 (0.83-16.62)	0.087	2.89 (0.63-13.28)	0.174
**RFS**					
	H-*GNA15*	2.36 (1.01-5.52)	0.047	2.45 (1.04-5.73)	0.040
	Age≥60 years	1,51 (0.50-4.53)	0.466		
	Female	0.39 (0.17-0.90)	0.028	0.37 (0.16-0.88)	0.024
	Risk group				
	Low *vs* High	0.92 (0.28-3.03)	0.889		
	Medium *vs* High	1.12 (0.44-2.85)	0.811		
	MRD	1.01 (0.99-1.03)	0.412		
	FLT3-ITD+	0.90 (0.33-2.45)	0.838		
	WT1	1.01 (0.99-1.03)	0.555		
	NPM1	0.96 (0.28-3.24)	0.940		
	DNMT3A	0.73 (0.17-3.13)	0.670		
	IDH1/2	1.10 (0.40-2.97)	0.864		
	TET2	1.25 (0.54-2.92)	0.603		
	RUNX1	2.23 (0.28-17.47)	0.446		

OS, overall survival; RFS, relapse free survival; H-GNA15, high GNA15 expression; L-GNA15, low GNA15 expression; HR, hazard ratio; CI, confidence interval; MRD, minimal residual disease.

### Down-Regulation of *GNA15* Inhibits Cell Proliferation and Colony Formation in AML Cells

**Figure 3:**
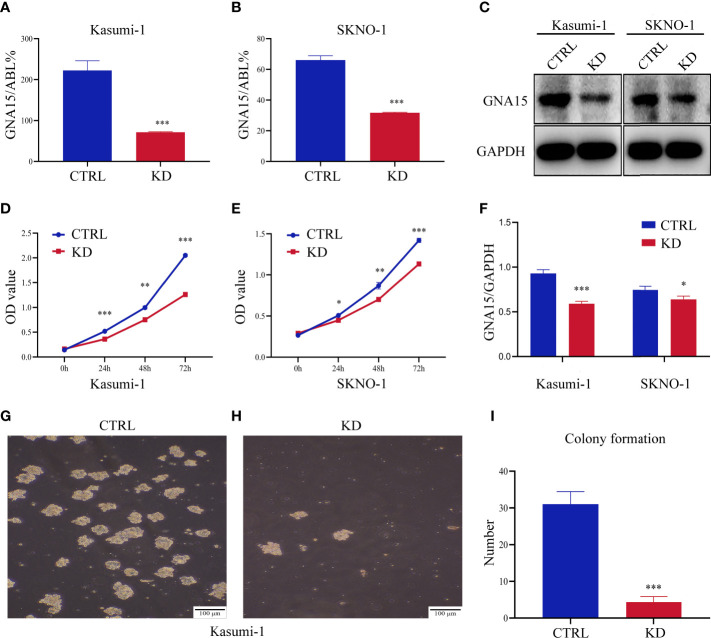
Down-regulation of *GNA15* inhibited cell proliferation and colony formation in AML cells. The efficiency of *GNA15* knockdown in the Kasumi-1 and SKNO-1 cell lines was verified by **(A, B)** real-time quantitative polymerase chain reaction and **(C, F)** Western blot, respectively. **(D, E)** Cell proliferation was detected by the CCK-8 assay in Kasumi-1 and SKNO-1**. (G–I)** The colony formation of *GNA15*-CTRL and *GNA15*-KD in Kasumi-1. CTRL, control; KD, knockdown; *P < 0.05 compared with CTRL cells; **P < 0.01 compared with CTRL cells; ***P < 0.001 compared with CTRL cells; Error bars indicate the standard deviation.

We quantified the transcriptional level and protein level of GNA15 in cell lines of hematological malignancies. RT-qPCR and Western blot analysis revealed that GNA15 is highly expressed in nearly all acute leukemia cell lines tested while lowly expressed in lymphoma and myeloma cell lines and normal controls (**Supplementary Table 2** and **Supplementary Figure 2**). Cell lines with stable *GNA15* knockdown and control cell lines using lentiviral small hairpin RNAs were constructed in Kasumi-1 and SKNO-1, and the knockdown efficiency was assessed by RT-qPCR (**Figures 3A, B**) and Western blot (**Figures 3C, F**). CCK8 analysis revealed a significant reduction in cell viability in both Kasumi-1 and SKNO-1 following *GNA15* knockdown (**Figures 3D, E**). Moreover, the knockdown of *GNA15* dramatically inhibited the cell colony formation ability in Kasumi-1 (**Figures 3G–I**). SKNO-1 control and knockdown cells failed to complete the colony formation test.

### Knockdown of *GNA15* Promotes Cell Cycle Arrest and Induces Apoptosis of AML Cells

**Figure 4:**
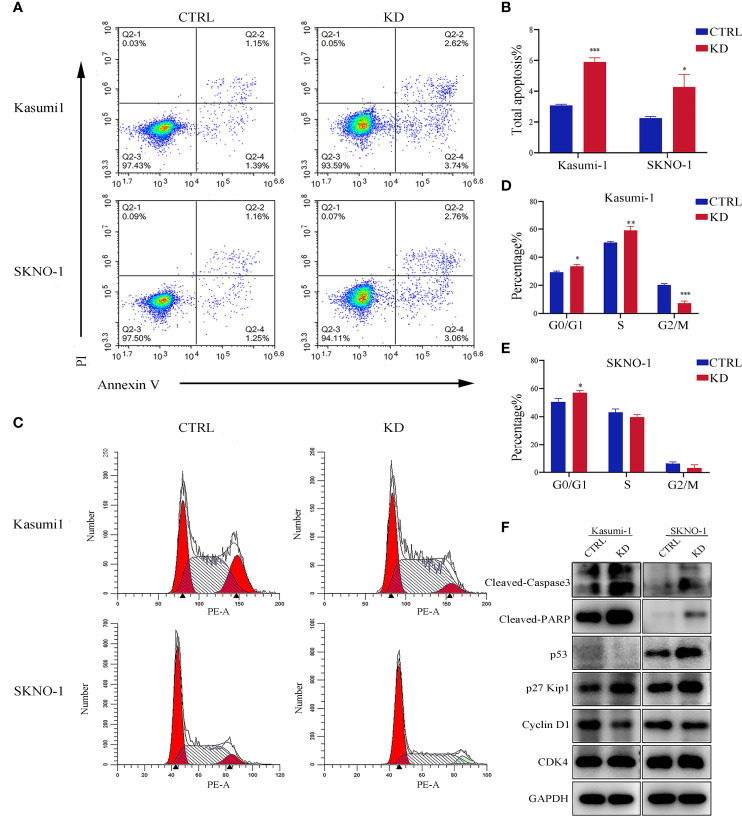
Apoptosis and cell cycle analysis in AML cell lines. **(A, B)** The flow cytometry was utilized to analyze apoptosis in *GNA15* knockdown cells compared with control. **(C–E)** Cell cycle was determined with propidium iodide (PI) staining in CTRL and KD cells. **(F)** Alterations in apoptosis and cell cycle-related protein assay in *GNA15*-KD cells compared to those of their vector controls. CTRL, control; KD, knockdown; *P < 0.05 compared with CTRL cells; **P < 0.01 compared with CTRL cells; ***P < 0.001 compared with CTRL cells.

Flow cytometry analysis showed that knockdown of *GNA15* significantly increased the apoptosis rate in both Kasumi-1 and SKNO-1 cells (**Figures 4A, B**). In addition, compared to the control group, knockdown of *GNA15* significantly increased cell counts in the G0/G1 phase in both Kasumi-1 and SKNO-1 and decreased cell counts in the G2/M phase in Kasumi-1(**Figures 4C–E**). To further explore the effect of *GNA15* knockdown on cell apoptosis and cell cycle, Western blot was used to evaluate proteins related to cell cycle and apoptosis. Knockdown of *GNA15* resulted in an increase in cleaved-PARP, cleaved-Caspase3, p53 and p27 and a significant decrease in Cyclin D1 but no change in CDK4 (**Figure 4F**).

### P38 MAPK Pathway Is Critical for *GNA15*-mediated Changes

**Figure 5:**
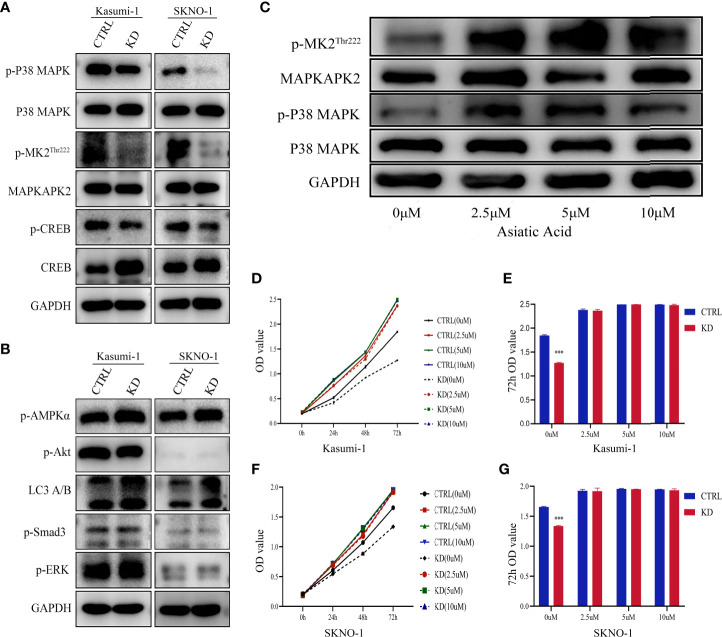
P38 MAPK pathway is critical for GNA15-mediated alterations. **(A)** Western blot analysis of P38 MAPK pathway in GNA15-KD cells and controls. **(B)** Western blot analysis of other proliferation- and metabolism-related proteins in GNA15-KD cells and controls. **(C)** The upregulation of P38 MAPK signaling pathway by Asiatic Acid (P38 MAPK activator) in GNA15-KD cells of Kasumi-1. **(D, F)** Asiatic Acid (2.5μM, 5μM, 10μM) reversed the inhibitory effect of GNA15 knockdown on the proliferation of Kasumi-1 **(D)** and SKNO-1 **(F)**. **(E, G)** Asiatic Acid (2.5μM, 5μM, 10μM) reversed the inhibitory effect of GNA15 knockdown on the proliferation of Kasumi-1 **(E)** and SKNO-1 **(G)** at 72h. CTRL, control; KD, knockdown; p-MK2^Thr222^: p-MAPKAPK2^Thr222^; ***P < 0.001 compared with CTRL cells; Error bars indicate the standard deviation.

To understand the mechanism behind the reduction in cell proliferation of *GNA15* knockdown, we examined the expression levels of phosphorylated proteins related to proliferation, metabolism, and autophagy. As shown in **Figure 5**, phosphorylation of P38 MAPK, MAPKAPK2^Thr222^ and CREB were notably decreased in *GNA15*-KD cells whereas phosphorylation of AMPK, Akt, Smad3, ERK and LC3 A/B were not significantly different (**Figures 5A, B**). To confirm the role of the P38 MAPK pathway in *GNA15*-mediated effects, we used Asiatic Acid, a P38 MAPK activator in *GNA15*-KD cells of Kasumi-1. As shown in **Figure 5C**, the addition of the P38 MAPK activator (2.5μM, 5μM, 10μM for 48 hours) could visibly increase the protein level of p-P38MAPK and the downstream p-MAPKAPK2^Thr222^. Phosphorylation of P38 MAPK after adding Asiatic Acid reversed the inhibitory effect of *GNA15* knockdown on the proliferation of AML cells (**Figures 5D–G**).

### Knockdown of *GNA15* Inhibits the Growth of Tumor Tissues *In Vivo*

**Figure 6:**
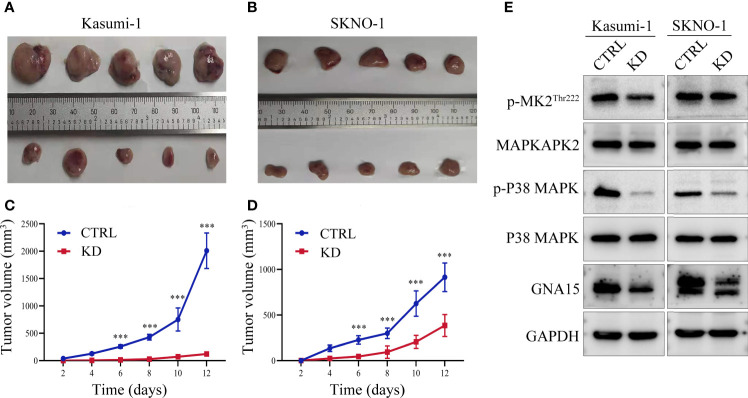
*GNA15* knockdown reduces the proliferation ability of AML cells in nude mice by inhibiting the P38 MAPK signaling pathway. **(A–D)** *GNA15*-KD inhibits tumor growth in Kasumi-1 and SKNO-1 cells. **(E)** The expression of P38 MAPK signaling pathway proteins in tumor tissues by Western blot. CTRL, control; KD, knockdown; ***P < 0.001 compared with CTRL cells. Error bars indicate the standard deviation.

Xenograft tumor model was used to verify the results of functional experiments and changes in signaling pathways *in vitro*. Our study showed that in Kasumi-1 cells, *GNA15* knockdown significantly inhibited the growth of tumors compared to controls (**Figures 6A, C**). The inhibitory effect was also verified in SKNO-1 cells (**Figures 6B, D**). Western blot was performed to investigate the mechanism of change, GNA15, p-P38 MAPK, p-MAPKAPK2^Thr222^ were significantly decreased (**Figure 6E**). These changes in the signaling pathway were the same as the results *in vitro*.

## Discussion

In this study, we evaluated the role of *GNA15* in predicting the prognosis of adult AML patients and investigated its molecular mechanism. Based on the bioinformatics analyses and our data, we found that the expression level of *GNA15* in AML was significantly higher than that of normal controls. The high expression of *GNA15* was independently associated with worse survival in AML with normal karyotype. *GNA15* knockdown inhibited cell proliferation and colony formation, induced cell apoptosis, and promoted cell cycle arrest in AML cell lines, and decreased cell proliferation through inhibiting the P38 MAPK pathway.

There are few studies on *GNA15*, and its effect on cancer was reported even less frequently. A study based on bioinformatics analysis suggested that *GNA15* may be related to ovarian cancer, but it has not been clinically and experimentally verified (16). The high expression of *GNA15* was associated with poor survival in small intestinal neuroendocrine neoplasia (8). de Jonge et al. reported that *GNA15* may be a specific marker of AML leukemia stem cells based on gene expression profile analysis of bone marrow CD34(+) and CD34(-) cells of AML patients and normal bone marrow CD34(+) cells (9). Their study suggested that *GNA15* can be used as a new marker for prognostic evaluation of AML with normal karyotype. However, the prognostic study of this study was based solely on the GEO database. Thus the prognostic significance of *GNA15* has not been verified in clinical samples, and the function and mechanism of *GNA15* in AML have not yet been studied.

In our study, we found that the expression of *GNA15* in acute leukemia was significantly higher than that in controls, and the expression of *GNA15* in AML was significantly higher than that in ALL. In addition, we found that the expression of *GNA15* in AML patients with *FLT3-ITD* (FMS-like tyrosine kinase-3 internal tandem duplications) was significantly higher than those without *FLT3-ITD*. Patients with *FLT3-ITD* account for 20% to 30% of AML (20), and often have a poor prognosis (21–23). Therefore, patients with high *GNA15* expression are more likely to have *FLT3-ITD* mutation, which may be one of the reasons for the adverse outcomes. There was no significant difference in the survival analysis of *GNA15* expression in the entire cohort, which may be caused by the complicated prognostic analysis of AML. For example, the application of hematopoietic stem cell transplantation, targeted drugs and immunotherapy could greatly improve the prognosis of high-risk patients (24, 25). Therefore, we further performed the prognostic analysis in adult AML with normal karyotype and found that high *GNA15* expression was independently associated with poor OS and RFS in this subgroup, which was consistent with the survival analysis results of the GEO database. It is worth noting that *FLT3-ITD* mutations, which constitute well-established negative prognostic factor, did not show significant correlation with OS and RFS. In our NK-AML cohort, there are 11 cases with *FLT3-ITD*, 4 of them had *NPM1* co-mutation and 2 of them received an allo-transplant. That may partially explain why *FLT3-ITD* mutations did not show significant correlation with OS and RFS. In order to solve the possible confounding factor of *FLT3-ITD mutations*, we performed a subgroup analysis of NK-AML without *FLT3-ITD* mutations and found that subjects with high *GNA15* expression also showed a worse OS in NK-AML without *FLT3-ITD* mutations. These results indicate that *GNA15* may be used as a potential marker for predicting survival and recurrence of AML patients, especially for patients with normal karyotype.

*GNA15* participates in a variety of biological processes through interactions with a diverse set of cellular targets (26). Zanini et al. showed that down-regulation of *GNA15* significantly inhibited cell proliferation in small intestinal neuroendocrine neoplasia, and the expression levels of cell cycle-dependent kinase inhibitors p16 and p21 were significantly reduced (8). In this study, we also found that the down-regulation of *GNA15* significantly inhibited proliferation and colony formation of AML cell lines, an increased proportion of apoptotic cells, and inhibited cell cycle progression. These results indicate that *GNA15* plays an oncogene role in AML and is likely to become the potential therapeutic target of AML.

To further explore the mechanism of the biological function, we studied the changes in important signaling pathways related to proliferation, metabolism and autophagy in AML cell lines. Mitogen-activated protein kinase (MAPK) is one of the main signaling pathways mediating the growth and survival of tumor cells and plays important roles in regulating cell proliferation, cell cycle, apoptosis, migration and invasion (27–29). Our results revealed that the phosphorylated P38 MAPK was significantly reduced in the *GNA15*-KD cells, while the phosphorylated ERK, AMPKα, Smad3, Akt and LC3 A/B showed no change. The results indicated that the P38 MAPK pathway was involved in inhibiting the proliferation of AML mediated by *GNA15* knockdown. MAPK-activated protein kinase (MAPKAPK2) is a target of P38 MAPK and acts directly after phosphorylation of P38 MAPK, under stress conditions (30, 31). cAMP-response element binding protein (CREB) is a bZIP transcription factor that can be activated by phosphorylated kinases such as MAPKAPK2, and then it activates the target gene through cAMP response elements to further mediate many physiological signaling transmission and regulate various cellular responses (32–34). Our research found that *GNA15* knockdown inhibited the expression of p-P38 MAPK and its downstream p- MAPKAPK2 and p-CREB, confirming that *GNA15* knockdown inhibits the activation of the p38 MAPK pathway. Moreover, a MAPK activator, Asiatic Acid, increased the protein level of p-P38MAPK and the downstream p- MAPKAPK2^Thr222^, which in turn ameliorated the suppressed proliferation caused by *GNA15* knockdown. The xenograft tumor mouse model also showed that *GAN15* affects the growth of tumor tissues *in vivo* by regulating the P38 MAPK pathway. These results suggest that *GNA15* could modulate the growth and survival of AML cells by regulating the P38 MAPK pathway.

There are several limitations to our study. Firstly, our study was retrospective, and the inherent biases should not be neglected. The second limitation is the small sample size, especially in the subgroup analysis. Also, there is the potential for an interaction between *GNA15* transcript levels and gene mutation including *FLT3-ITD*. Due to these limitations, our present conclusions need to be further validated in prospectively randomized studies with large samples.

## Conclusions

In summary, our research indicated that *GNA15* was highly expressed in adult AML, and the high expression of *GNA15* was independently correlated with worse OS and RFS in adult AML with normal karyotype. In addition, *GNA15* knockdown inhibited cell proliferation, promoted cell cycle arrest, and induced apoptosis of AML cells. Moreover, GNA15 modulated the growth of AML cells by regulating the P38 MAPK signaling pathway. Although the detailed mechanisms still require further investigation, our current research suggests *GNA15* may serve as a potential prognostic marker and a therapeutic target for AML in the future.

## Data Availability Statement

Publicly available datasets were analyzed in this study. This data can be found here: http://immuco.bjmu.edu.cn https://www.oncomine.org.

## Ethics Statement

The studies involving human participants were reviewed and approved by the Ethics Committee of the First Affiliated Hospital of Zhengzhou University. Written informed consent for participation was not required for this study in accordance with the national legislation and the institutional requirements. The animal study was reviewed and approved by the Ethics Committee of the Zhengzhou University Animal Center.

## Author Contributions

Conceptualization and design: CW, SW, and YFL. Data acquisition: ML, YL, LY, YX, and WW. Methodology: SW and LY. Data analysis and interpretation: ML, CW, and SW; Writing (original draft): ML. Writing (review and editing): SW, CW, and YJL. Project administration: CW, SW, and ZJ.

## Funding

This work was supported by the National Natural Science Foundation of China [grant number 81800137 and U1804191] and Henan Medical Science and Technique Foundation (grant number 2018020068).

## Conflict of Interest

The authors declare that the research was conducted in the absence of any commercial or financial relationships that could be construed as a potential conflict of interest.

## Publisher’s Note

All claims expressed in this article are solely those of the authors and do not necessarily represent those of their affiliated organizations, or those of the publisher, the editors and the reviewers. Any product that may be evaluated in this article, or claim that may be made by its manufacturer, is not guaranteed or endorsed by the publisher.

## Abbreviations

AML, acute myeloid leukemia; BM, bone marrow; CCK8, Cell Counting Kit-8; CREB, cAMP-response element binding protein; DEG, differentially expressed gene; DAVID, The Database for Annotation, Visualization and Integrated Discovery; FLT3-ITD, FMS-like tyrosine kinase-3 internal tandem duplications; GNA15, guanine nucleotide binding protein subunit alpha-15; GEO, Gene Expression Omnibus; GO, gene ontology; HSC, hematopoietic stem cell; IST, in silico transcriptomics database; KEGG, Kyoto Encyclopedia of Genes and Genomes; LSC, leukemia stem cell; MAPK, mitogen-activated protein kinase; MAPKAPK2, MAPK-activated protein kinase 2; OS, overall survival; PBMC, peripheral blood mononuclear cell; RFS, relapse-free survival; RT-qPCR, reverse transcription-quantitative polymerase chain reaction.
