# Supplementary material for: Downregulation of GNA15 Inhibits Cell Proliferation via P38 MAPK Pathway and Correlates with Prognosis of Adult Acute Myeloid Leukemia With Normal Karyotype
Source: Front Oncol. 2021 Sep 6;11:724435. doi: 10.3389/fonc.2021.724435 (PMC8451478; doi:10.3389/fonc.2021.724435)
Supplement: Supplementary file 1 [file DataSheet_1.docx]

Supplementary Material

Downregulation of GNA15 inhibits cell proliferation via P38 MAPK Pathway and Correlates with Prognosis of Adult Acute Myeloid Leukemia with Normal Karyotype

Mengya Li ^1^, Yu Liu ^1^, Yajun Liu ^2^, Lu Yang ^1^, Yan Xu ^1^, Weiqiong Wang ^1^, Zhongxing Jiang ^1^, Yanfang Liu^1^, Shujuan Wang ^1*^ , Chong Wang ^1*^

**Supplementary figures:**


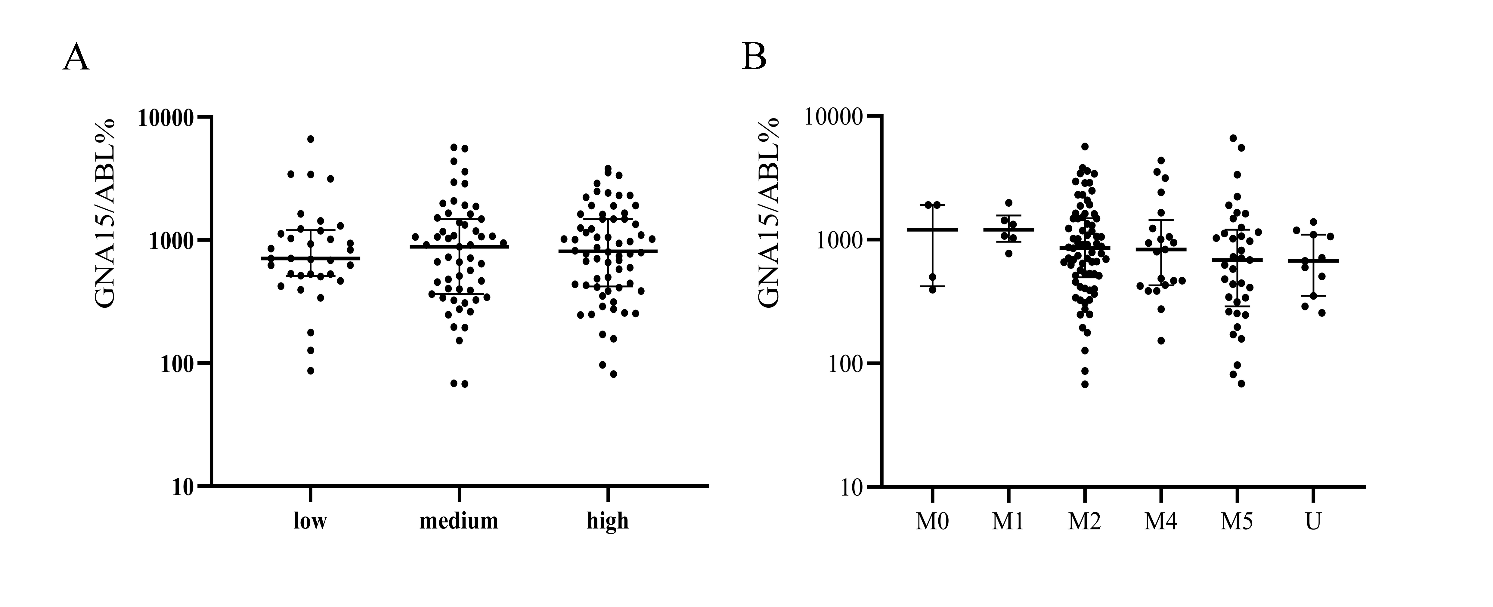
**Figure S1. (A)** The transcript levels of *GNA15* in 154 AML patients according to cytogenetic risk stratifications. **(B)** The transcript levels of *GNA15* in 154 AML patients according to French-America-British (FAB) classification.


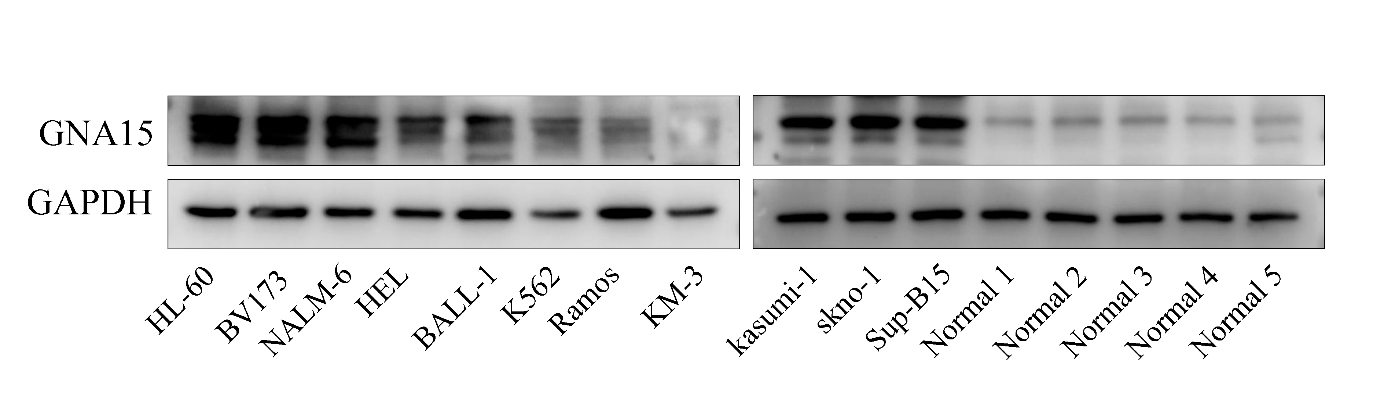


**Figure S2.** Western blot analysis of GNA15 in acute leukemia, lymphoma and myeloma cell lines and normal controls.

**Supplementary tables:**

| **TABLE S1 ∣**Sequences of primers and probes used in this study | |
| --- | --- |
| Name | Sequence (5'-3') |
| GNA15-Forward primer | CTACCAGAACATCTTCGTGTCCAT |
| GNA15-Reverse primer | GCTGAATCGAGCAGGTGGAA |
| GNA15-Probe | FAM-TACCCTGGTTCAAAAGCACATCCGTCAT-BHQ |
| ABL-Forward primer | CCGCTGACCATCAATAAGGAA |
| ABL-Reverse primer | GATGTAGTTGCTTGGGACCCA |
| ABL-Probe | FAM-CCATTTTTGGTTTGGGCTTCACACCATT-TAMARA |

| **TABLE S2∣**Tanscript Levels of GNA15 in Cell Lines. | | | | |
| --- | --- | --- | --- | --- |
| Name of cells | Type of cells | | Repeating times | GNA15/ABL1%^a^ |
| KASUMI-1 | Human acute myeloid leukemia | | 3 | 207.47±59.61 |
| SKNO-1 | Human acute myeloid leukemia | | 3 | 290.13±76.57 |
| HL-60 | Human acute promyelocytic leukemia | | 3 | 129.04±41.10 |
| HEL | Human erythrocyte leukemia | | 3 | 84.35±16.35 |
| Sup-B15 | Human acute B lymphoblastic leukemia | | 3 | 301.59±29.26 |
| BV173 | Human acute B lymphoblastic leukemia | | 3 | 85.25±15.30 |
| NALM-6 | Human acute B lymphoblastic leukemia | | 3 | 116.15±22.42 |
| BALL-1 | Human acute B lymphoblastic leukemia | | 3 | 0.28±0.02 |
| K562 | Human chronic myeloid leukemia | 3 | | 0.12±0.04 |
| Ramos | Human Burkitt lymphoma | 3 | | 1.66±0.09 |
| KM-3 | Human multiple myeloma | 3 | | 0.14±0.06 |
| *a.Mean±SD* |  |  | |  |
